# Identification of Dietary Pattern Networks Associated with Gastric Cancer Using Gaussian Graphical Models: A Case-Control Study

**DOI:** 10.3390/cancers12041044

**Published:** 2020-04-23

**Authors:** Madhawa Gunathilake, Jeonghee Lee, Il Ju Choi, Young-Il Kim, Jeongseon Kim

**Affiliations:** 1 Department of Cancer Control and Population Health, Graduate School of Cancer Science and Policy, Goyang-si 10408, Gyeonggi-do, Korea; 1806104@ncc.re.kr; 2 Department of Cancer Biomedical Science, Graduate School of Cancer Science and Policy, Goyang-si 10408, Gyeonggi-do, Korea; jeonghee@ncc.re.kr; 3 Center for Gastric Cancer, National Cancer Center Hospital, National Cancer Center, Goyang-si 10408, Gyeonggi-do, Korea; cij1224@ncc.re.kr (I.J.C.); 11996@ncc.re.kr (Y.-I.K.)

**Keywords:** Gaussian graphical models, dietary patterns, gastric cancer, networks

## Abstract

Gaussian graphical models (GGMs) are novel approaches to deriving dietary patterns that assess how foods are consumed in relation to one another. We aimed to apply GGMs to identify dietary patterns and to investigate the associations between dietary patterns and gastric cancer (GC) risk in a Korean population. In this case-control study of 415 GC cases and 830 controls, food intake was assessed using a 106-item semiquantitative food frequency questionnaire that captured 33 food groups. The dietary pattern networks corresponding to the total population contained a main network and four subnetworks. For the vegetable and seafood network, those who were in the highest tertile of the network-specific score showed a significantly reduced risk of GC both in the total population (OR = 0.66, 95% CI = 0.47–0.93, *p* for trend = 0.018) and in males (OR = 0.55, 95% CI = 0.34–0.89, *p* for trend = 0.012). Most importantly, the fruit pattern network was inversely associated with the risk of GC for the highest tertile (OR = 0.56, 95% CI = 0.38–0.81, *p* for trend = 0.002). The identified vegetable and seafood network and the fruit network showed a protective effect against GC development in Koreans.

## 1. Introduction

Gastric cancer (GC) has been identified as the fifth most common cancer type and is one of the main causes of cancer-related death worldwide [1]. The GC incidence in eastern Asia, including Korea, is the highest worldwide; it is over four times higher than the rates in Western Europe [2]. It has been reported that the age-adjusted incidence rate of GC is 34.0 per 100,000 in Korea [3]. According to a prediction of cancer incidence and mortality in Korea, the incidence of stomach cancer increases gradually with age for both sexes [4].

Several lifestyle factors, particularly dietary factors, can influence the risk of GC [5]. The need to address the relationship between diet and disease has driven several investigations, the majority of which mostly focused on a single nutrient or food item [6]. It is a well-known that studies focused on the relationship between a single nutrient/food item and disease have significantly advanced knowledge of the etiology and prevention of several diseases, particularly cancer [7]. However, it is important to note that focusing on a single nutrient or a food item to observe associations has some conceptual and methodological challenges [6]. Generally, humans consume nutrients as part of a meal, and those nutrients have synergistic metabolic actions in the body; thus, differentiating their specific effects is complicated [8].

To address these challenges, a dietary pattern approach has been suggested as a complementary strategy for investigating diet and disease relationships [9]. This approach addresses dietary intake as a pattern rather than as a sum of single nutrients consumed together [9]. Dietary patterns can be defined as the quantities, proportions, varieties or combinations of different foods and nutrients in diets and the frequency with which they are habitually consumed [10]. Dietary patterns have been accepted as a favorable method for optimizing dietary intake and explaining the complexity of eating behaviors. Several approaches have been proposed for deriving dietary patterns, and exploratory analyses based on data-reduction methods, such as principal component analysis (PCA) and reduced rank regression (RRR), are commonly used to derive dietary patterns [6].

Innovative methods for deriving dietary patterns, such as Gaussian graphical models (GGMs), have been applied as exploratory approaches by addressing pairwise correlations between two food variables while controlling for the indirect effects of the other food variables [11]. As GGMs are a type of graphical method, identified internal patterns can be represented as networks, and those networks may identify key interrelated food groups that may be potential candidates for further investigations of relationships between diet and disease. Most importantly, the food group present in the original data set is part of only one network at a time. In contrast to existing approaches, such as PCA, one or more food groups may be significantly correlated with more than one pattern based on the component loadings. Thus, it is important to note that GGMs can be considered an innovative exploratory approach for deriving dietary patterns [11].

In the present study, we aimed to use GGMs to identify dietary pattern networks associated with GC based on the partial correlations of the food groups. Furthermore, we aimed to investigate the associations between the identified dietary pattern networks and GC risk in a Korean population.

## 2. Results

### 2.1. General Characteristics

Table 1 presents the general characteristics of the study participants with and without GC. The proportion of current smokers was higher in the case group (30.8%) than in the control group (19.5%), whereas the proportion of nonsmokers was lower in the case group (40.2%) than in the control group (46.3%) (*p* < 0.001). The proportion of nondrinkers was similar in the case group (28.7%) and the control group (28.4%). The case group was more likely to have a family history of GC (*p* < 0.001). Patients in the case group engaged in less regular exercise (*p* < 0.001), were less educated (*p* < 0.001), and exhibited lower employment rates (*p* < 0.001) and lower monthly incomes (*p* < 0.001) than those in the control group. The proportion of *Helicobacter pylori* (*H. pylori*) seropositivity in the case group (92.1%) was higher than that in the control group (58.6%). Men and women in the control group had higher education levels, had higher monthly incomes, were more likely to be employed, were less likely to be smokers, engaged in more regular exercise, and had a lower proportion of *H. pylori* infection than those in the case group.

### 2.2. Comparison of the Intake of Food Groups

Table 2 shows the comparisons of mean intakes of the food groups between the case and control groups estimated from the semiquantitative food frequency questionnaire (SQFFQ). There were significantly higher intakes of refined grains and kimchi in the case group than in the control group. Intakes of tubers and roots, bread, legumes, tofu/soymilk, nuts and seeds, processed meat, poultry, eggs, milk, fruit, fruit products, green/yellow vegetables, light-colored vegetables, mushrooms, condiments/seasonings, and coffee/tea were significantly higher in the control group than in the GC case group for the whole study population.

Regarding the male population, refined grain intake was significantly higher in the case group than in the control group, whereas intakes of tubers and roots, bread, legumes, meat byproducts, poultry, eggs, milk, fruit, fruit products, green/yellow vegetables, light-colored vegetables, mushrooms, condiments/seasonings and coffee/tea intakes were significantly higher in the control group than in the case group. For females, refined grain intake was significantly higher in the case group than in the control group, whereas the intake of rice cakes, bread, legumes, nuts and seeds, processed meat, eggs, dairy products, fruit, fruit products, and condiments/seasonings were significantly higher in the control group than the case group (Table 2).

### 2.3. Dietary Pattern Networks Derived by GGMs

**Figure 1:**
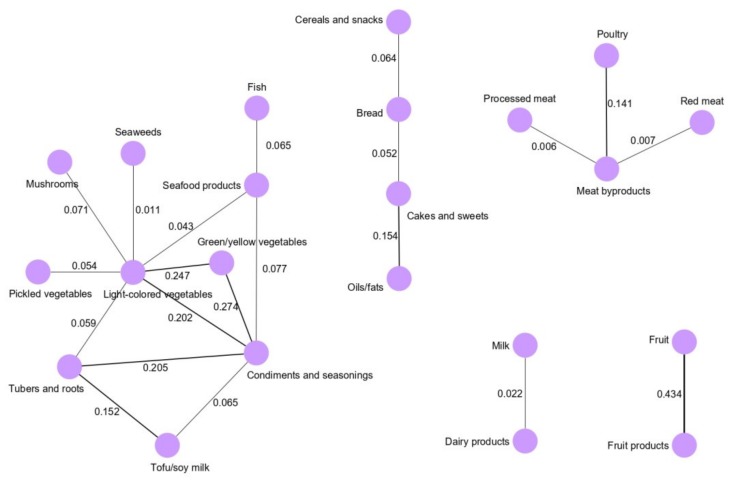
Dietary intake networks for whole study population derived by Gaussian graphical models.

Dietary pattern networks were derived for the whole study population and for each sex. Figure 1 shows the dietary pattern networks derived by GGMs for the whole study population. The dietary pattern networks corresponding to the total population contain a main network and four subnetworks. The main dietary network (the vegetables and seafood pattern) was composed of 10 food groups. Most of the food groups in the vegetable and seafood network were clustered around light-colored vegetables and condiments and seasonings. Light-colored vegetables, green/yellow vegetables, pickled vegetables, tubers and roots, mushrooms, tofu/soy milk, seaweeds, fish, seafood products, and condiments and seasonings were clustered in the vegetable and seafood network. In particular, condiments and seasonings were highly correlated with green/yellow vegetables (0.274), light-colored vegetables (0.202) and tubers and roots (0.205). Green/yellow vegetables and light-colored vegetables were correlated (0.247). Light-colored vegetables were correlated with seafood products (0.043) and seaweeds (0.011). The other four subnetworks identified for the whole population were the snack and fat network, meat network, dairy network and fruit network. In the fruit network, fruit and fruit products were conditionally dependent and strongly correlated (0.434).

**Figure 2:**
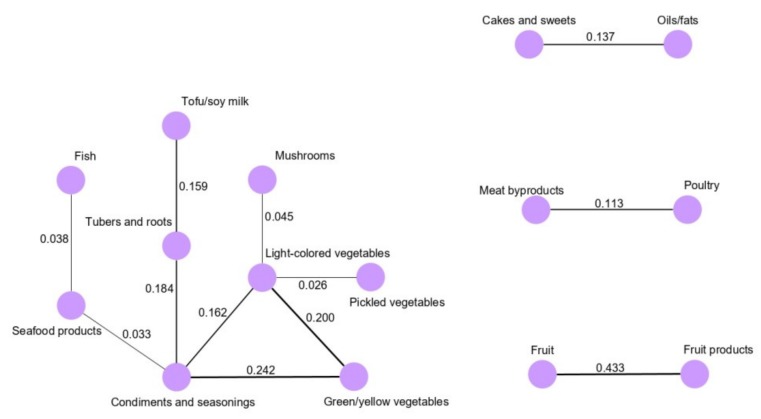
Dietary intake networks derived by Gaussian graphical models for the male population.

Regarding the dietary networks derived for the male population, four patterns were identified (Figure 2). The vegetable and seafood network was the main network, while the other three subnetworks were the snacks and fats network, meat network and fruit network. In the vegetables and seafood network, green/yellow vegetables (0.242), light-colored vegetables (0.162), and tubers and roots (0.184) were highly correlated with condiments and seasonings. Additionally, condiments and seasonings intake was correlated with seafood products (0.033), and seafood product intake was correlated with fish (0.038). In the snacks and fats network, cake and sweets intake was correlated with oils/fats (0.137). Meat byproduct and poultry intakes were conditionally dependent in the meat network (0.113), whereas fruit and fruit products were strongly correlated in the fruit network (0.433).

**Figure 3:**
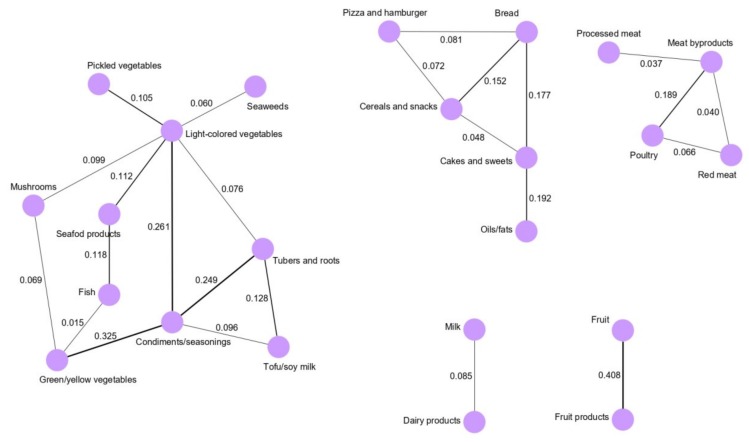
Dietary intake networks derived by Gaussian graphical models for the female population.

For the females, five dietary networks were derived: the vegetable and seafood network, snacks and fats network, meat network, dairy network, and fruit network (Figure 3). The vegetable and seafood network consisted of 10 food groups, which were mainly clustered around the light-colored vegetable and condiment/seasoning intakes. Light-colored vegetable intake was highly correlated with condiments and seasonings (0.261), pickled vegetables (0.105) and seafood products (0.112). Additionally, condiment and seasoning intake was correlated with green/yellow vegetable (0.325) and tuber and root intakes (0.249). In the snacks and fats network, bread was correlated with cereals and snacks (0.152) and cake and sweets (0.177). Additionally, pizza and hamburger intake was correlated with bread intake (0.081) in the female population. In the meat network, processed meat, red meat, and poultry intakes were clustered around meat byproducts intake, and meat byproducts were highly correlated with poultry (0.189). In the dairy network, milk and dairy products were correlated (0.085), whereas in the fruit network, fruit and fruit products were strongly correlated (0.408).

Regarding the dietary networks derived for the intestinal type of GC, five patterns were identified (Figure S1). The vegetable and seafood network was the main network, and four subnetworks were the snacks and fats network, meat network, dairy network and fruit network. In the vegetables and seafood network, light-colored vegetables (0.239) and condiments/seasonings (0.289) were highly correlated with green/yellow vegetables. Tubers and roots were correlated with the tofu/soy milk (0.153), and condiments/seasonings (0.195), and light-colored vegetables (0.069). Interestingly, condiments/seasonings were negatively correlated with tofu/soy milk (−0.021). In the snacks and fat network, cake and sweets intake was correlated with oils/fats (0.150). Meat byproduct and poultry intakes were conditionally dependent in the meat network (0.126), whereas fruit and fruit products were strongly correlated in the fruit network (0.425).

Figure S2 represents the five dietary pattern networks derived for the diffuse type of GC. The vegetable and seafood network was the main network, while the other four subnetworks were the snacks and fats network, meat network, dairy network and fruit network. In the vegetables and seafood network, green/yellow vegetables (0.241), condiments/seasonings (0.190), tubers and roots (0.052), pickled vegetables (0.057), tofu/soy milk (0.016), and mushrooms (0.077) were correlated, and the cluster was centered around light-colored vegetables. Green/yellow vegetables and condiments/seasonings were highly correlated (0.276). In the snacks and fats network, cake and sweets intake was correlated with oils/fats (0.147). Meat by product and poultry intakes were conditionally dependent in the meat network (0.138), whereas fruit and fruit products were strongly correlated in the fruit network (0.425).

### 2.4. Association Between GGM-Derived Dietary Pattern Networks and GC Risk

Table 3 shows the ORs and 95% CIs according to the network-specific score tertiles for each dietary pattern network for the whole study population.

In the vegetable and seafood network, those who were in the highest tertile of the network-specific score showed a significantly reduced risk of GC in model II (OR = 0.66, 95% CI = 0.47–0.93, *p* for trend = 0.018) compared to those in the lowest tertile. Most importantly, the fruit pattern network was inversely associated with the risk of GC in the highest vs the lowest tertile (OR = 0.54, 95% CI = 0.38–0.77, *p* for trend < 0.001 in model II and OR = 0.56, 95% CI = 0.38–0.81, *p* for trend = 0.002 in model III).

Table 4 presents the ORs and 95% CIs according to the network-specific score tertiles for each dietary pattern network for the male and female populations. Regarding the male population, those in the highest tertile of the network-specific score for the vegetables and seafood pattern showed a significantly reduced risk of GC compared to those in the lowest tertile in models II and III (OR = 0.51, 95% CI = 0.32–0.81, *p* for trend = 0.003 and OR = 0.55, 95% CI = 0.34–0.89, *p* for trend = 0.012, respectively).

For the female population, those who were in the middle tertile of the network—specific score for the fruit network showed a significantly reduced risk of GC (OR = 0.54, 95% CI = 0.32–0.93). However, those who were in the highest tertile of the network-specific score showed a marginally significant decreasing association with GC risk (OR = 0.56, 95% CI = 0.32–1.00), and there was a borderline significant linear trend (*p* = 0.064) in model II. However, none of the association results were significant in the other dietary pattern networks (Table 4).

Table S1 shows the ORs and 95% CIs according to the network—specific score tertiles for each dietary pattern for the intestinal type of GC. For the vegetable and seafood network, those who were in the highest tertile of the network-specific score showed a significantly reduced risk of GC in model III (OR = 0.52, 95% CI = 0.30–0.91, *p* for trend = 0.021) compared to those in the lowest tertile. Most importantly, the fruit pattern network was inversely associated with the risk of GC in the highest vs the lowest tertile (OR = 0.50, 95% CI = 0.29–0.87, *p* for trend = 0.014 in model II and OR = 0.53, 95% CI = 0.30–0.93, *p* for trend = 0.027 in model III). Table S2 shows the results for the association between the dietary pattern networks derived from GGMs and the risk of the diffuse type of GC. The fruit pattern network was inversely associated with the risk of GC in the highest vs the lowest tertile (OR = 0.55, 95% CI = 0.34–0.89, *p* for trend = 0.016 in model II and OR = 0.55, 95% CI = 0.33–0.92, *p* for trend = 0.019 in model III).

Table S3 shows the interaction between the GGM derived dietary patterns and sex for the risk of GC. In males, those who were in the third tertile of the vegetable and seafood network-specific score showed a significantly reduced risk of GC compared to those in the lowest tertile in model II (OR: 0.55, 95% CI: 0.34–0.89). However, significant interactions between dietary patterns and sex were not observed.

## 3. Discussion

In this case-control study, GGMs derived a main dietary pattern network, which was the vegetable and seafood network, and four additional four subnetworks. The vegetable and seafood network consisted of green/yellow vegetables, light-colored vegetables, pickled vegetables, tubers and roots, tofu/soy milk, condiments and seasonings, seafood products, seaweeds, and fish. This indicates that the foods in the vegetables and seafood network were conditionally independent of any specific food group of other networks. The vegetable and seafood network and fruit network were associated with a decreased risk of GC for the whole study population. GGMs are a powerful exploratory method for dietary pattern analysis [11]. Theoretically, GGMs assess pairwise correlations between two food groups, and this helps to elucidate how different food groups are consumed in relation to one another. According to the conditional independence theory, the use of partial correlation coefficients evaluates the association between two food groups independent of the effects of other food groups [11,12].

We identified a main dietary pattern network for the whole study population, that was composed mainly of vegetable and seafood food groups. Basically, the vegetable, mushrooms, tofu/soymilk, tubers and roots, condiments and seasonings, and seafood food groups were conditionally dependent on the main dietary pattern network, indicating that dietary behavior among Koreans mainly involves vegetables and seafood items. In addition, four subnetworks were obtained as dietary pattern networks for the whole study population. In the snacks and fats pattern network, cereals and snacks, bread, cakes and sweets and oils/fats were correlated with the other food groups. This dietary pattern network represents snacking behavior in the Korean diet. Most importantly, milk and milk products were correlated in the dairy network, whereas fruit and fruit products were highly correlated in the fruit network.

A study conducted using the European Prospective Investigation into Cancer and Nutrition (EPIC)-Potsdam cohort showed that two main dietary networks were derived for males and females separately [11]. Both networks were basically composed of intakes of red meat, processed meat, cooked vegetables, sauces, potatoes, cabbage, poultry, legumes, mushrooms, soups, whole grains and refined breads. Regarding the comparison of this result with the current study findings, it is notable that the dietary patterns and dietary behaviors are totally dependent on ethnicity and that each ethnicity has unique dietary behaviors [13]. Furthermore, direct comparison of the patterns may not be ideal because of the differences in methodological approaches [12]. As a consequence, it may be important to consider the components of identified patterns when making comparisons.

It is important to observe the association between derived dietary pattern networks and health outcomes, and it helps to have a clear understanding of the diet and disease relationship to be compared [12]. In the current study, we observed that those in the highest tertile of the vegetable and seafood network-specific score had a reduced risk of GC compared to those in the lowest tertile. It is obvious that the combination of vegetables and seafood represents a traditional Korean dietary behavior that is a healthy pattern for Koreans. A study revealed that a prudent/healthy pattern played a favorable role in GC development whereas a Western/unhealthy pattern played an unfavorable role in GC development [14]. A recent EPIC-Potsdam study observed that GGMs identified food intake networks, and the risk analysis of noncommunicable diseases revealed that increased adherence to the GGM Western-type pattern was associated with an increased risk of type 2 diabetes in women, whereas adherence to a high-fat dairy pattern was associated with a decreased risk of type 2 diabetes in both men and women [12]. Furthermore, a study based on a Portuguese urban population confirmed that the derived fruit and vegetables pattern had a protective effect against GC risk [15].

We observed that those who were in the highest tertile of the fruit pattern network-specific score showed a significantly reduced risk of GC compared to those in the lowest tertile for the whole population and for males. A study carried out in Japan focusing on dietary patterns and stomach cancer among middle-aged male workers concluded that vegetable and fruit consumption patterns were negatively associated with GC [16]. The World Cancer Research Fund/American Association for Cancer Research stated that the intake of fruits is a convincing protective factor against GC [5]. Fruits are mainly associated with antioxidant-related nutrients, such as vitamin C and carotenoids, and such nutrients might have a protective effect against GC development [17,18]. A population-based case-control study conducted in Sweden reported that a healthy dietary pattern characterized by the consumption of vegetables, tomatoes, fruit, fish and poultry moderately reduced the risk of gastric cardia adenocarcinoma, while a Western dietary pattern was associated with an increased risk of gastric cardia adenocarcinoma [19]. In contrast, a prospective study concluded that a healthy dietary pattern decreased the risk of GC in females, while the traditional dietary pattern increased the risk of GC in both sexes in Japan [20]. A possible reason for the increased risk of GC associated with traditional dietary patterns is the high intake of pickled vegetables, salted fish and miso soup; these food items can increase the GC risk through exposure to genotoxic markers [20].

A study conducted in Korea using the Cancer Screening Examination Cohort to identify the major dietary patterns in Korean adults found four major dietary patterns, namely ”rice and kimchi”, “vegetables and fish”, ”fruits and dairy”, and ”meats and sweets” [21]. The study observed that the fruit and dairy pattern was a protective factor against the gastrointestinal cancers and concluded that the traditional Korean dietary pattern composed of rice, kimchi, soybean paste and vegetables may decrease the cancer risk in Korean adults [21]. A Korean study conducted to observe the dietary patterns associated with colorectal cancer identified three main dietary patterns, namely, traditional, Western and prudent, using PCA [22]. Both the traditional pattern and the prudent pattern were inversely associated with colorectal cancer risk. The Western pattern was positively associated with cancer, especially in females [22]. Although the studies were not related to GC, the Korea National Health and Nutrition Examination Surveys (KNHNES) from 2008 to 2011 observed that a vegetable and fish dietary pattern was positively associated with skeletal muscle mass in Korean men [23]. A systematic review and meta-analysis of the associations of dietary intake with cardiovascular disease in a Korean population summarized that adherence to a healthy dietary pattern (a rice-based or traditional pattern) showed borderline relationships with a decreased risk of elevated total cholesterol and elevated triglycerides [24]. Another study carried out using KNHNES data from 2008 to 2010 to observe the association between dietary patterns and hypertension among Korean adults identified three major dietary patterns in both sexes, namely, ”traditional”, ”Western” and ”dairy and carbohydrates” [25]. They concluded that the dairy and carbohydrate pattern was inversely associated with hypertension prevalence among Korean adults [25].

It is well known that there are several approaches for deriving dietary patterns and that those approaches have been applied in nutritional epidemiology research [9]. A population-based case-control study conducted in a high-risk area in Central Italy used factor analysis and multiple correspondence analysis to derive dietary patterns and found that two patterns, named “traditional” and “vitamin-rich”, were strongly associated with GC risk and overall accounted for 44% of the estimated attributable risk of GC. The other two patterns, “refined” and “fat-rich”, were not consistently associated with GC [26]. A study conducted in Mexico used factor analysis to derive the dietary patterns associated with GC [27]. They found three major dietary patterns in the factor analysis, and the first dietary pattern, characterized by vegetables, fruits, and white meat components, was significantly associated with a reduced risk of GC. Another study applied principal component and cluster analysis to derive dietary patterns associated with GC and found three dietary patterns, namely, high consumption of fruits and dairy products and low consumption of alcoholic beverages; low consumption of fruit, salads, vegetables, dairy products, fish and meat; and high consumption of most food groups and low vegetable soup intake. The pattern of low consumption of fruit, salads, vegetables, dairy products, fish and meat was significantly associated with an increased risk of GC [15].

We identified the vegetable and seafood dietary pattern as the main pattern for the whole study population and sex-specific populations. Interestingly, most of the food groups identified in the main networks in our study had similarities to the food groups included in the healthy or prudent dietary patterns identified in previous studies [19,20]. Traditional Korean foods are considered healthy for Koreans, yet GC is a common type of cancer in Korea. It is important to note that the International Agency for Research on Cancer has classified *H. pylori* infection as a group 1 carcinogen in humans [28]. It is a well-known fact that *H. pylori* infection is common in East Asian countries, including Korea. It has been reported that the *H. pylori* infection was responsible for 80.3% of noncardia GC in men and 78.7% in women [29]. Thus, it might be one reason why GC is common in Korea. In addition, high salt consumption is associated with the risk of GC specifically in Korea [30]. It has been noted that Koreans consume significantly high amount of salts due to the frequent ingestion of foods such as kimchi and soy bean paste that evidently increase the risk of GC by causing direct damaging to the stomach lining, increasing the formation of nitroso compounds, and facilitating *H. pylori* infection [29].

The main strength of our study was the methodological approach that employed GGMs to identify conditional independence among food intake variables that could not be addressed by other data-reduction approaches, such as PCA or RRR. Second, GGMs minimize the subjective choices during data analysis; consequently, the results are robust enough to interpret. Third, GGMs introduce sparsity, which helps to identify the most important food variables to be included in the final model. Fourth, the use of GGMs to derive dietary pattern networks leads to a much better understanding of the biological relations between diet and health status. Finally, the identified networks can be converted into a specific score to describe the association between derived dietary patterns and GC risk.

Certain limitations of the current study need to be considered. First, the underlying data need to follow a Gaussian distribution, which is not the case for all dietary variables. Thus, a method that can ensure a normal distribution of the variables, such as log transformation, should be applied. Second, as our study was a case-control study, it was prone to recall bias, and cancer patients were more likely than healthy controls to recall unhealthy dietary habits. Third, the naming of the identified dietary pattern network is dependent on the researcher, which may cause some problems in comparing results.

## 4. Materials and Methods

### 4.1. Study Population

This study is an extension of previously published case-control studies [17,18,31,32,33,34,35,36,37,38,39,40]. Participants were recruited from the National Cancer Center Hospital in Korea between March 2011 and December 2014. Individuals who had been histologically confirmed as having early GC within the preceding three months at the Center for Gastric Cancer were included in the case group. Early GC was defined as an invasive carcinoma confined to the mucosa and/or submucosa, regardless of lymph node metastasis status [41]. Patients diagnosed with diabetes mellitus, a history of cancer within the past five years, advanced GC, or severe systemic or mental disease, as well as women who were pregnant or breastfeeding, were excluded. The control subjects were selected from health-screening examinations performed at the Center for Cancer Prevention and Detection at the same hospital. Individuals with a history of cancer, diabetes mellitus, gastric ulcers, and *H. pylori* treatment were excluded from the control group. In total, 1727 participants were recruited (1227 controls and 500 cases), and 1671 individuals provided data through an SQFFQ and a self-administered questionnaire. Individuals with a total energy intake of <500 kcal or ≥4000 kcal (n = 15) were excluded because of the reliability of the data. Of the 1656 participants remaining, the control and case groups were frequency-matched by age (within five years) and sex at a ratio of 2:1 (controls: cases). The final sample included 1245 participants comprising 830 controls and 415 cases (men, 810; women, 435). This study was approved by the Institutional Review Board of the National Cancer Center (IRB Number: NCCNCS-11-438). Written informed consent was obtained from all participants.

### 4.2. Data Collection

The participants were asked to complete a self-administered questionnaire. Demographic, lifestyle, and medical history data were collected from the participants. Total energy intake was obtained from the SQFFQ, which has been previously reported to be a reliable and valid questionnaire [42]. The SQFFQ includes nine food consumption frequency categories (i.e., never or rarely, once a month, two or three times a month, once or twice a week, three or four times a week, five or six times a week, once a day, twice a day, and three times a day) and three portion-size categories (i.e., small, medium, and large) for specific food items consumed within the past 12 months. The average daily nutrient intake for each participant was calculated using CAN-PRO 4.0 (Computer Aided Nutritional Analysis Program, Korean Nutrition Society, Seoul, Korea).

### 4.3. Statistical Analysis

#### 4.3.1. Demographic and Dietary Intake Assessments

To compare the demographic and lifestyle characteristics between the controls and cases, chi-square and Student’s *t*-tests were performed for categorical variables and continuous variables, respectively. The 106 food items listed in the SQFFQ were collapsed into 33 food groups according to their culinary use and nutrient profile. The means ± standard deviations (SDs) of food group intakes were calculated, and the mean dietary intakes were compared between the controls and cases using Student’s *t*-test.

#### 4.3.2. Assessment of Dietary Patterns by GGMs

GGMs were used to derive dietary pattern networks using dietary intake variables. The theoretical background of GGMs can be found elsewhere [11]. In principle, suppose there is a data matrix (X) with *n* observations and *p* variables that has a mean vector µ and covariance matrix Σ. The conditional distribution of any two random variables given other variables can be obtained from the inverse of the covariance matrix (precision matrix). The correlation coefficient in this distribution between two variables is called the partial correlation. If the partial correlation between two variables given the rest of the variables is zero, it can be inferred that these two variables are conditionally independent. The basis of GGMs is the estimation of the conditional independence of the inverse of the covariance matrix, and it can be reflected in an undirected graph. In a high-dimensional, multivariate, normally distributed data set, there may be no or few zero entries in the precision matrix. This results in a fully connected graph in which each node is connected to other nodes in the graph. Such a concentrated graph is less informative since the aim of GGMs is to identify the internal structure of the graphical model which is an accurate representation of the underlying data. The accuracy of such a model is assessed by the likelihood that the model explains the data. This requires a regularization technique that enforces sparsity in the precision matrix. Graphical lasso has been identified as a fast and efficient approach for performing the regularization. It puts a penalty on the off-diagonal elements of the precision matrix, shrinking the estimated values of pairwise partial correlations, which forces small or noisy values to zero and results in a sparse matrix. Regularization is achieved by penalizing the log likelihood by the term λ × L1 norm, where L1 is the absolute sum of the inverse covariance matrix, and λ is the nonnegative-tuning shrinkage parameter, which is also known as the regularization parameter. The λ depends on the research question and is estimated using the best model fit for a series of λ values [11].

First, the food intake variables were checked for normal distribution using histograms to address the Gaussian assumption. Since most of the dietary intake variables had a skewed distribution, the intake variables were log transformed [log_10_ (g/d + 1)] to improve normality. Dietary intake data were converted into a data matrix, and the inverse covariance matrix was obtained. A series of regularization parameter λ values was obtained using the “huge” package [43]. The ranges of the λ values decreased from 0.81 to 0.08 for the whole study population. A sparse inverse covariance (precision) matrix was obtained by using the graphical lasso (least absolute shrinkage and selection operator) with the optimum λ set as 0.38 by using “glasso” R package [44]. Then, the dietary pattern network was obtained with respect to the precision matrix by using the R “qgraph” package. Finally, the precision matrix was imported to yEd graph editor and visualized as a dietary network [45]. The analysis was carried out separately for men and women to observe the sex-specific networks.

#### 4.3.3. Association between GGM-Identified Networks and GC Risk

The strength of each node in the identified network was calculated in terms of its centrality value in the network theory. Then, we combined the strength of each node with the dietary intake data to calculate a network-specific score for each study participant. The network (pattern) scores were used as exposure variables for further association analysis. The network-specific scores were categorized into tertiles according to the distribution of the controls. The lowest tertile of the pattern score was used as the reference group. The odds ratio (ORs) and 95% confidence intervals (CIs) were estimated using unconditional logistic regression models. The median values of the network scores in each category were used as continuous variables to test for trends. OR estimates were calculated for three models: model I was the crude model; model II was adjusted for age, sex, family history of GC, smoking status, regular exercise, education, occupation, income and total energy intake; and model III was additionally adjusted for *H. pylori* infection status. Association analysis was performed for men and women separately. The test for interaction between dietary pattern networks and sex in relation to GC was conducted using logistic regression models via likelihood ratio tests. All statistical analyses were carried out using SAS version 9.4 software (SAS, Inc., Cary, NC, USA) and the R platform (version 3.5.1) (The R Foundation for Statistical Computing, Vienna, Austria).

## 5. Conclusions

In conclusion, this study showed that the GGMs identified that the vegetable and seafood network was associated with a reduced risk of GC for the whole population and the male population. Moreover, the fruit pattern network was significantly associated with a reduction in GC risk for the whole study population, indicating that fruit consumption behavior has a remarkable effect on the reduction of GC risk among Koreans. However, additional studies to validate this methodological approach in other populations are needed.

## Figures and Tables

**Table 1:** General characteristics of the study population.

	All (n = 1245)			Male (n = 810)			Female (n = 435)		
Variable	Controls (n = 830)	Cases (n = 415)	*p*-value ^b^	Controls ((n = 540)	Cases (n = 270)	*p*-value ^b^	Controls (n = 290)	Cases (n = 145)	*p*-value ^b^
Age (y)	53.7 ± 9.0	53.8 ± 9.3	0.89	54.8 ± 8.4	54.9 ± 8.7	0.91	51.6 ± 9.8	51.7 ± 9.9	0.94
<50	285 (34.3)	139 (33.5)	0.78	153 (28.33)	77 (28.5)	0.96	132 (45.5)	62 (42.8)	0.59
≥50	545 (65.7)	276 (66.5)		387 (71.7)	193 (71.5)		158 (54.5)	83 (57.2)	
Sex [n (%)]			0.99						
Male	540 (65.1)	270 (65.1)							
Female	290 (34.9)	145 (34.9)							
Body mass index (kg/m^2^) [n (%)]	23.9 ± 2.9	23.9 ± 3.0	0.63	24.4 ± 2.7	24.2 ± 3.0	0.39	23.1 ± 3.1	23.2 ± 3.0	0.79
<23	314 (37.8)	159 (38.3)	0.98	161 (29.8)	91 (33.7)	0.51	153 (52.8)	68 (46.9)	0.53
23–25	249 (30.0)	122 (29.4)		170 (31.5)	78 (28.9)		79 (27.2)	44 (30.3)	
≥25	266 (32.1)	133 (32.1)		209 (38.7)	101 (37.4)		57 (19.7)	32 (22.1)	
Missing	1 (0.1)	1 (0.2)		0 (0.0)	0 (0.0)		1 (0.3)	1 (0.7)	
Smoking status [n (%)]			<0.001			<0.001			0.021
Current smoker	162 (19.5)	128 (30.8)		157 (29.1)	121 (44.8)		5 (1.7)	7 (4.8)	
Ex-smoker	284 (34.2)	119 (28.7)		277 (51.3)	110 (40.7)		7 (2.4)	9 (6.2)	
Non-smoker	384 (46.3)	167 (40.2)		106 (19.6)	39 (14.4)		278 (95.9)	128 (88.3)	
Missing	0 (0.0)	1 (0.2)		0 (0.0)	0 (0.0)		0 (0.0)	1 (0.7)	
Alcohol consumption [n (%)]			0.24			0.28			0.82
Current drinker	534 (64.3)	254 (61.2)		404 (74.8)	193 (71.5)		130 (44.8)	61 (42.1)	
Ex-drinker	60 (7.2)	41 (9.9)		47 (8.7)	33 (12.2)		13 (4.5)	8 (5.5)	
Non-drinker	236 (28.4)	119 (28.7)		89 (16.5)	44 (16.3)		147 (50.7)	75 (51.7)	
Missing	0 (0.0)	1 (0.2)		0 (0.0)	0 (0.0)		0 (0.0)	1 (0.7)	
First-degree family history of gastric cancer			<0.001			0.003			0.11
Yes	103 (12.4)	82 (19.8)		74 (13.7)	60 (22.2)		29 (10.0)	22 (15.2)	
No	725 (87.4)	332 (80.0)		464 (85.9)	209 (77.4)		261 (90.0)	123 (84.8)	
Missing	2 (0.2)	1 (0.2)		2 (0.4)	1 (0.4)		0 (0.0)	0 (0.0)	
Regular exercise [n (%)]			<0.001			<0.001			<0.001
Yes	466 (56.1)	147 (35.4)		303 (56.1)	109 (40.4)		163 (56.2)	38 (26.2)	
No	361 (43.4)	268 (64.6)		234 (43.3)	161 (59.6)		127 (43.8)	107 (73.8)	
Missing	3 (0.4)	0 (0.0)		3 (0.6)	0 (0.0)		0 (0.0)	0 (0.0)	
Educational level [n (%)]			<0.001			<0.001			<0.001
Middle school	119 (14.3)	142 (34.2)		71 (13.2)	91 (33.7)		48 (16.6)	51 (35.2)	
High school	253 (30.5)	174 (41.9)		140 (25.9)	112 (41.5)		113 (38.9)	62 (42.8)	
College or more	426 (51.3)	97 (23.4)		301 (55.7)	66 (24.4)		125 (43.1)	31 (21.4)	
Missing	32 (3.9)	2 (0.5)		28 (5.2)	1 (0.4)		4 (1.4)	1 (0.7)	
Occupation [n (%)]			<0.001			0.009			0.002
Group 1: Professional, administrative management	156 (18.8)	70 (16.9)		117 (21.7)	59 (21.9)		39 (13.5)	11 (7.6)	
Group 2: Office, sales and service positions	266 (32.1)	122 (29.4)		203 (37.6)	81 (30.0)		63 (21.7)	41 (28.3)	
Group 3: Agriculture, laborer	128 (15.4)	104 (25.1)		111 (20.6)	83 (30.7)		17 (5.9)	21 (14.5)	
Group 4: Unemployed and other	277 (33.4)	117 (28.2)		106 (19.6)	46 (17.0)		171 (58.9)	71 (49.0)	
Missing	3 (0.4)	2 (0.5)		3 (0.6)	1 (0.4)		0 (0.0)	1 (0.7)	
Marital status [n (%)]			0.61			0.48			0.98
Married	716 (86.3)	361 (87.0)		478 (88.5)	243 (90.0)		238 (82.1)	118 (81.4)	
Other (single, divorced, separated, widowed, cohabitating)	113 (13.6)	52 (12.5)		61 (11.3)	26 (9.6)		52 (17.9)	26 (17.9)	
Missing	1 (0.1)	2 (0.5)		1 (0.2)	1 (0.4)		0 (0.0)	1 (0.7)	
Monthly income [n (%)] ^a^			<0.001			<0.001			0.016
<200	149 (18.0)	133 (32.1)		85 (15.7)	85 (31.5)		64 (22.1)	48 (33.1)	
200–400	341 (41.1)	148 (35.7)		232 (43.0)	106 (39.3)		109 (37.6)	42 (28.9)	
≥400	273 (32.9)	96 (23.1)		168 (31.1)	55 (20.4)		105 (36.2)	41 (28.3)	
Missing	67 (8.1)	38 (9.2)		55 (10.2)	24 (8.9)		12 (4.1)	14 (9.7)	
*H. pylori* infection			<0.001			<0.001			<0.001
Positive	486 (58.6)	382 (92.1)		333 (61.7)	252 (93.3)		153 (52.8)	130 (89.7)	
Negative	320 (38.6)	33 (8.0)		187 (34.6)	18 (6.7)		133 (45.9)	15 (10.3)	
Missing	24 (2.9)	0 (0.0)		20 (3.7)	0 (0.0)		4 (1.4)	0 (0.0)	
Total energy intake (Kcal/day)	1713.6 ± 545.5	1924.1 ± 612.9	<0.001	1760.6 ± 541.5	2038.5 ± 634.8	<0.001	1626.0 ± 543.1	1711.1 ± 507.0	0.12

Values are expressed as mean ± SD or n (%).^a^ Unit is 10,000 Won in Korean currency. ^b^ *p*-values present the difference between cases and controls. Age, body mass index (continuous), and total energy intake were examined using Student’s t-tests; other variables were assessed using chi-square analysis.

**Table 2:** Dietary intakes of the 33 food groups used to derive the dietary networks. ^a^

	Total Population			Male Population			Female Population		
Food Group (g/Day)	Controls (n = 830)	Cases (n = 415)	*p*-Value ^b^	Controls (n = 540)	Cases (n = 270)	*p*-Value ^b^	Controls (n = 290)	Cases (n = 145)	*p*-Value ^b^
Refined grains	548.8 ± 179.6	602.6 ± 168.4	<0.001	578.1 ± 166.7	610.0 ± 168.2	0.011	491.1 ± 189.9	588.7 ± 168.4	<0.001
Whole grains	7.56 ± 6.61	7.13 ± 6.45	0.28	7.42 ± 6.79	6.69 ± 6.49	0.15	7.82 ± 6.27	7.95 ± 6.32	0.84
Tubers and roots	48.09 ± 48.51	41.23 ± 40.14	0.008	42.53 ± 38.92	34.39 ± 37.36	0.005	58.44 ± 61.32	53.95 ± 42.11	0.37
Noodles	48.11 ± 50.45	45.08 ± 43.52	0.27	52.25 ± 52.77	46.74 ± 42.95	0.11	40.41 ± 44.92	41.99 ± 44.56	0.73
Rice cakes	15.42 ± 41.95	11.30 ± 40.86	0.10	11.31 ± 33.19	8.89 ± 44.41	0.43	23.07 ± 53.87	15.77 ± 32.95	0.08
Bread	41.71 ± 130.70	21.22 ± 68.17	<0.001	32.25 ± 108.6	15.79 ± 39.79	0.002	59.32 ± 162.8	31.31 ± 101.2	0.03
Cereals and snacks	11.76 ± 69.50	7.40 ± 37.86	0.15	6.18 ± 26.07	6.20 ± 41.05	0.99	22.15 ± 111.4	9.63 ± 31.06	0.08
Pizza, hamburgers	12.44 ± 54.79	15.80 ± 122.9	0.60	8.55 ± 37.34	7.57 ± 55.67	0.79	19.68 ± 77.01	31.12 ± 193.0	0.49
Cakes and sweets	12.26 ± 14.53	11.74 ± 15.44	0.56	12.19 ± 15.50	12.05 ± 16.67	0.90	12.36 ± 12.52	11.16 ± 12.86	0.35
Legumes	5.41 ± 10.19	3.84 ± 6.73	0.001	4.95 ± 9.36	3.79 ± 6.74	0.04	6.27 ± 11.55	3.92 ± 6.73	0.008
Tofu/soymilk	59.20 ± 74.65	50.81 ± 51.88	0.021	57.68 ± 75.25	48.79 ± 53.31	0.05	62.01 ± 73.56	54.56 ± 49.06	0.21
Nuts and seeds	4.34 ± 11.17	2.20 ± 5.58	<0.001	2.97 ± 6.59	2.08 ± 5.85	0.05	6.91 ± 16.32	2.41 ± 5.04	<0.001
Red meat	54.21 ± 36.03	52.79 ± 33.23	0.50	56.18 ± 34.88	55.25 ± 31.90	0.71	50.56 ± 37.86	48.23 ± 35.24	0.54
Meat byproducts	10.65 ± 66.16	5.89 ± 35.97	0.10	9.92 ± 66.34	3.60 ± 10.39	0.031	12.01 ± 65.94	10.14 ± 59.08	0.77
Processed meat	4.91 ± 24.94	1.62 ± 5.93	<0.001	2.78 ± 10.17	1.66 ± 6.51	0.06	8.88 ± 39.58	1.55 ± 4.64	0.002
Poultry	9.85 ± 23.19	7.35 ± 17.22	0.033	8.35 ± 17.95	5.84 ± 12.58	0.021	12.63 ± 30.50	10.17 ± 23.35	0.35
Fish	21.44 ± 20.85	20.24 ± 17.37	0.28	21.60 ± 21.02	20.78 ± 17.19	0.55	21.14 ± 20.58	19.22 ± 17.69	0.32
Seafood and seafood products	18.45 ± 15.33	19.24 ± 21.22	0.50	17.50 ± 11.92	19.87 ± 24.63	0.14	20.22 ± 20.11	18.08 ± 12.60	0.18
Seaweeds	2.04 ± 1.79	2.02 ± 1.89	0.78	1.89 ± 1.75	1.78 ± 1.62	0.36	2.32 ± 1.82	2.45 ± 2.26	0.55
Eggs	19.27 ± 17.85	15.00 ± 17.13	<0.001	18.53 ± 17.29	14.33 ± 16.95	0.001	20.64 ± 18.79	16.26 ± 17.46	0.019
Milk	260.60 ± 1009.0	141.20 ± 647.3	0.012	175.1 ± 853.8	82.32 ± 368.3	0.032	419.8 ± 1233.4	250.7 ± 965.7	0.12
Dairy products	71.99 ± 235.6	51.59 ± 276.5	0.20	65.54 ± 270.1	48.61 ± 330.9	0.468	84.03 ± 151.6	57.14 ± 123.2	0.05
Fruit	156.00 ± 173.1	110.70 ± 134.6	<0.001	122.5 ± 132.9	94.98 ± 125.3	0.005	218.4 ± 216.7	140.1 ± 146.3	<0.001
Fruit products	36.87 ± 50.82	25.03 ± 40.27	<0.001	30.28 ± 43.92	20.03 ± 29.22	<0.001	49.14 ± 59.82	34.33 ± 54.16	0.01
Green/yellow vegetables	95.41 ± 78.40	82.32 ± 64.71	0.002	86.62 ± 76.04	76.11 ± 57.62	0.028	111.8 ± 80.23	93.89 ± 75.03	0.03
Light-colored vegetables	86.59 ± 59.87	78.69 ± 51.24	0.016	83.23 ± 53.06	74.04 ± 47.52	0.013	92.85 ± 70.51	87.36 ± 56.68	0.38
Pickled vegetables	4.11 ± 8.52	4.22 ± 8.64	0.84	3.91 ± 7.34	4.35 ± 8.28	0.46	4.49 ± 10.38	3.96 ± 9.28	0.61
Kimchi	145.6 ± 105.7	161.5 ± 131.8	0.033	148.6 ± 106.7	161.1 ± 131.4	0.18	139.9 ± 103.9	162.3 ± 133.0	0.08
Mushrooms	9.25 ± 11.39	7.56 ± 9.41	0.005	7.58 ± 8.48	6.24 ± 6.47	0.013	12.35 ± 14.95	10.01 ± 12.93	0.09
Oils/fats	4.80 ± 4.76	4.67 ± 5.55	0.68	5.09 ± 4.73	5.23 ± 6.18	0.75	4.26 ± 4.76	3.62 ± 3.89	0.14
Condiments/seasonings	17.79 ± 13.74	15.54 ± 10.64	0.002	17.12 ± 13.69	14.98 ± 10.15	0.013	19.03 ± 13.75	16.56 ± 11.46	0.049
Carbonated beverages	135.2 ± 1170.7	124.9 ± 1259.5	0.89	134.1 ± 1157.3	168.6 ± 1554.5	0.75	137.3 ± 1197.3	43.53 ± 189.7	0.19
Coffee/tea	68.40 ± 108.4	51.29 ± 103.3	0.007	60.16 ± 87.46	43.22 ± 95.77	0.012	83.75 ± 138.2	66.34 ± 115.0	0.17

^a^ Values are presented in means ± SD. ^b^ *p*-values were obtained using Student’s *t*-test. The listed 33 food groups were collapsed from the 106-item SQFFQ based on culinary usage and similar nutrient profiles.

**Table 3:** Association between dietary pattern networks derived from GGMs and GC risk for the whole population.

Dietary Patterns	No. of Controls	No. of Cases	Model I OR (95% CI)	Model II OR (95% CI)	Model III OR (95% CI)
Vegetables and seafood					
T1 (low)	276 (33.3)	165 (39.8)	1.00	1.00	1.00
T2 (medium)	277 (33.4)	154 (37.1)	0.93 (0.71–1.23)	0.98 (0.72–1.34)	1.03 (0.74–1.44)
T3 (high)	277 (33.4)	96 (23.1)	0.58 (0.43–0.78)	0.66 (0.47–0.93)	0.72 (0.50–1.03)
*p* for trend			<0.001	0.018	0.07
Snacks and fats					
T1 (low)	276 (33.3)	171 (41.2)	1.00	1.00	1.00
T2 (medium)	278 (33.5)	144 (34.7)	0.84 (0.63–1.10)	0.89 (0.66–1.22)	1.03 (0.74–1.44)
T3 (high)	276 (33.3)	100 (24.1)	0.59 (0.43–0.79)	0.88 (0.61–1.27)	0.93 (0.64–1.37)
*p* for trend			<0.001	0.53	0.70
Dairy					
T1 (low)	276 (33.3)	197 (47.5)	1.00	1.00	1.00
T2 (medium)	277 (33.4)	125 (30.1)	0.63 (0.48–0.84)	0.77 (0.56–1.05)	0.68 (0.48–0.95)
T3 (high)	277 (33.4)	93 (22.4)	0.47 (0.35–0.63)	0.87 (0.60–1.26)	0.88 (0.59–1.31)
*p* for trend			<0.001	0.73	0.96
Meat					
T1 (low)	277 (33.4)	172 (41.5)	1.00	1.00	1.00
T2 (medium)	276 (33.3)	139 (33.5)	0.81 (0.61–1.07)	0.89 (0.64–1.24)	0.81 (0.57–1.14)
T3 (high)	277 (33.4)	104(25.1)	0.61 (0.45–0.81)	0.88 (0.59–1.31)	0.83 (0.55–1.28)
*p* for trend			0.001	0.59	0.54
Fruit					
T1 (low)	277 (33.4)	202 (48.7)	1.00	1.00	1.00
T2 (medium)	276 (33.3)	134 (32.3)	0.67 (0.51–0.88)	0.84 (0.62-1.15)	0.85 (0.61–1.18)
T3 (high)	277 (33.4)	79 (19.0)	0.39 (0.29-0.53)	0.54 (0.38–0.77)	0.56 (0.38–0.81)
*p* for trend			<0.001	<0.001	0.002

Model I: crude model; model II: adjusted for age, sex, family history of gastric cancer, smoking status, regular exercise, education, occupation, income and total energy intake; model III: additionally adjusted for *H. pylori* infection status.

**Table 4:** Association between dietary pattern networks derived from GGMs and GC risk in male and female populations.

Dietary Patterns	No. of Controls	No. of Cases	Model I OR (95% CI)	Model II OR (95% CI)	Model III OR (95% CI)
Males					
Vegetables and seafood					
T1 (low)	180 (33.3)	104 (38.5)	1.00	1.00	1.00
T2 (medium)	179 (33.2)	115 (42.6)	1.11 (0.79–1.56)	1.22 (0.82–1.80)	1.25 (0.82–1.91)
T3 (high)	181 (33.5)	51 (18.9)	0.48 (0.33–0.72)	0.51 (0.32–0.81)	0.55 (0.34–0.89)
*p* for trend			<0.001	0.003	0.012
Snacks and fats					
T1 (low)	179 (33.2)	99 (36.7)	1.00	1.00	1.00
T2 (medium)	180 (33.3)	100 (37.0)	1.00 (0.71–1.42)	1.07 (0.72–1.60)	1.03 (0.67–1.58)
T3 (high)	181 (33.5)	71 (26.3)	0.71 (0.49–1.03)	0.78 (0.50–1.20)	0.80 (0.50–1.28)
*p* for trend			0.05	0.22	0.31
Meat					
T1 (low)	180 (33.3)	119 (44.1)	1.00	1.00	1.00
T2 (medium)	180 (33.3)	82 (30.4)	0.69 (0.48–0.98)	0.84 (0.56–1.27)	0.93 (0.60–1.44)
T3 (high)	180 (33.3)	69 (25.6)	0.58 (0.40–0.83)	1.17 (0.72–1.90)	1.23 (0.74–2.06)
*p* for trend			0.01	0.34	0.33
Fruit					
T1 (low)	180 (33.3)	124 (45.9)	1.00	1.00	1.00
T2 (medium)	180 (33.3)	80 (29.6)	0.65 (0.46–0.91)	0.81 (0.54–1.21)	0.77 (0.50–1.19)
T3 (high)	180 (33.3)	66 (24.4)	0.53 (0.37–0.77)	0.77 (0.50–1.17)	0.76 (0.48–1.19)
*p* for trend			0.002	0.25	0.29
Females					
Vegetables and seafood					
T1 (low)	97 (33.5)	61 (42.1)	1.00	1.00	1.00
T2 (medium)	96 (33.1)	47 (32.4)	0.78 (0.48–1.25)	0.85 (0.50–1.45)	1.04 (0.58–1.84)
T3 (high)	97 (33.5)	37 (25.5)	0.61 (0.37–0.99)	0.76 (0.43–1.34)	0.82 (0.45–1.51)
*p* for trend			0.049	0.34	0.52
Snacks and fat					
T1 (low)	96 (33.1)	55 (37.9)	1.00	1.00	1.00
T2 (medium)	97 (33.5)	60 (41.4)	1.08 (0.68–1.71)	1.09 (0.65–1.85)	1.29 (0.73–2.27)
T3 (high)	97 (33.5)	30 (20.7)	0.54 (0.32–0.91)	0.62 (0.31–1.22)	0.65 (0.32–1.34)
*p* for trend			0.009	0.11	0.15
Meat					
T1 (low)	97 (33.5)	57 (39.3)	1.00	1.00	1.00
T2 (medium)	96 (33.1)	38 (26.2)	0.67 (0.41–1.11)	0.72 (0.40–1.30)	0.67 (0.36–1.27)
T3 (high)	97 (33.5)	50 (34.5)	0.88 (0.55–1.41)	0.85 (0.46–1.56)	0.65 (0.34–1.23)
*p* for trend			0.81	0.79	0.26
Dairy					
T1 (low)	97 (33.5)	61 (42.1)	1.00	1.00	1.00
T2 (medium)	97 (33.5)	50 (34.5)	0.82 (0.51–1.31)	0.94 (0.56–1.59)	0.89 (0.51–1.56)
T3 (high)	96 (33.1)	34 (23.5)	0.56 (0.34–0.93)	0.82 (0.43–1.57)	0.80 (0.40–1.59)
*p* for trend			0.032	0.57	0.57
Fruit					
T1 (low)	97 (33.5)	82 (56.6)	1.00	1.00	1.00
T2 (medium)	96 (33.1)	35 (24.1)	0.43 (0.27–0.70)	0.54 (0.32–0.93)	0.59 (0.33–1.05)
T3 (high)	97 (33.5)	28 (19.3)	0.34 (0.20–0.57)	0.56 (0.32–1.00)	0.62 (0.34–1.14)
*p* for trend			<0.001	0.06	0.15

Model I: crude model; model II: adjusted for age, family history of gastric cancer, smoking status, regular exercise, education, occupation, income and total energy intake; model III: additionally adjusted for *H. pylori* infection status.

## Supplementary Materials

The following are available online at https://www.mdpi.com/2072-6694/12/4/1044/s1, Figure S1: Dietary intake networks for intestinal type GC derived by Gaussian graphical models, Figure S2: Dietary intake networks for diffuse type GC derived by Gaussian graphical models, Table S1: Association between dietary pattern networks derived from GGMs and intestinal type of GC risk, Table S2: Association between dietary pattern networks derived from GGMs and diffuse type of GC risk, Table S3: Interaction between GGM derived dietary patterns and sex in the risk of GC.
