# Supplementary material for: Identification of Dietary Pattern Networks Associated with Gastric Cancer Using Gaussian Graphical Models: A Case-Control Study
Source: Cancers (Basel). 2020 Apr 23;12(4):1044. doi: 10.3390/cancers12041044 (PMC7226381; doi:10.3390/cancers12041044)
Supplement: Supplementary file 1 [file cancers-12-01044-s001.pdf]

Supplementary Material: Identification of Dietary Pattern Networks Associated with Gastric Cancer Using Gaussian Graphical Models: A Case-Control Study

Madhawa Gunathilake, Jeonghee Lee, Il Ju Choi, Young-Il Kim and Jeongseon Kim

Table S1. Association between dietary pattern networks derived from GGMs and intestinal type of GC risk.

Dietary patterns	No. of controls	No. of cases	Model I OR (95% CI)	Model II OR (95% CI)	Model III OR (95% CI)
Vegetables and seafood					
T1 (low)	276 (33.3)	69 (43.7)	1.00	1.00	1.00
T2 (medium)	277 (33.4)	57 (36.1)	0.82 (0.56–1.21)	0.84 (0.53–1.33)	0.86(0.53–1.38)
T3 (high)	277 (33.4)	32 (20.3)	0.46 (0.29–0.73)	0.47 (0.28–0.80)	0.52(0.30–0.91)
p for trend			<0.001	0.006	0.021
Snacks and fats					
T1 (low)	277 (33.4)	70 (44.3)	1.00	1.00	1.00
T2 (medium)	276 (33.3)	58 (36.7)	0.83 (0.56–1.22)	0.84 (0.54–1.32)	0.91 (0.57–1.45)
T3 (high)	277 (3.4)	30 (19.0)	0.43 (0.27–0.68)	0.59 (0.33–1.04)	0.60 (0.33–1.08)
p for trend			<0.001	0.070	0.084
Milk and dairy					
T1 (low)	276 (33.3)	78 (49.4)	1.00	1.00	1.00
T2 (medium)	276 (33.3)	51 (32.3)	0.65 (0.44–0.97)	0.89 (0.57–1.42)	0.85 (0.53–1.38)
T3 (high)	278 (33.5)	29 (18.4)	0.37 (0.23–0.58)	0.92 (0.50–1.66)	0.94 (0.51–1.76)
p for trend			<0.001	0.839	0.982
Meat					
T1 (low)	276 (33.3)	70 (44.3)	1.00	1.00	1.00
T2 (medium)	278 (33.5)	49 (31.0)	0.70 (0.47–1.04)	0.83 (0.51–1.35)	0.76 (0.46–1.26)
T3 (high)	276 (33.3)	39 (24.7)	0.56 (0.36–0.85)	1.17 (0.63–2.15)	1.07 (0.57–2.02)
p for trend			0.012	0.506	0.679
Fruit					
T1 (low)	276 (33.3)	85 (53.8)	1.00	1.00	1.00
T2 (medium)	277 (33.4)	48 (30.4)	0.56 (0.38–0.83)	0.79 (0.50–1.25)	0.72 (0.45–1.17)
T3 (high)	277 (33.4)	25 (15.8)	0.29 (0.18–0.47)	0.50 (0.29–0.87)	0.53 (0.30–0.93)
p for trend			<0.001	0.014	0.027

Model I: crude model; model II: adjusted for age, sex, family history of gastric cancer, smoking status, regular exercise, education, occupation, income and total energy intake; model III: additionally adjusted for *H. pylori* infection status

Table S2. Association between dietary pattern networks derived from GGMs and diffuse type of GC risk.

Dietary patterns	No. of controls	No. of cases	Model I OR (95% CI)	Model II OR (95% CI)	Model III OR (95% CI)
Vegetables and seafood					
T1 (low)	276 (33.3)	61 (37.2)	1.00	1.00	1.00
T2 (medium)	277 (33.4)	59 (36.0)	0.96 (0.65–1.43)	1.07 (0.69–1.66)	1.13 (0.72–1.80)
T3 (high)	277 (33.4)	44 (26.8)	0.72 (0.47–1.09)	0.80 (0.50–1.29)	0.86 (0.53–1.42)
p for trend			0.115	0.338	0.533
Snacks and fats					
T1 (low)	276 (33.3)	64 (39.0)	1.00	1.00	1.00
T2 (medium)	277 (33.4)	56 (34.2)	0.87 (0.58–1.30)	0.95 (0.61–1.47)	1.15 (0.72–1.82)
T3 (high)	277 (33.4)	44 (26.8)	0.68 (0.45–1.04)	0.98 (0.60–1.64)	1.11 (0.65–1.88)
p for trend			0.078	0.987	0.743
Milk and dairy					
T1 (low)	277 (33.4)	72 (43.9)	1.00	1.00	1.00
T2 (medium)	277 (33.4)	51 (31.1)	0.71 (0.48–1.05)	0.78 (0.50–1.21)	0.64 (0.40–1.02)
T3 (high)	276 (33.3)	41 (25.0)	0.57 (0.38–0.87)	0.94 (0.56–1.57)	0.89 (0.52–1.53)
p for trend			0.022	0.952	0.898
Meat					
T1 (low)	276 (33.3)	65 (39.6)	1.00	1.00	1.00
T2 (medium)	277 (33.4)	55 (33.5)	0.84 (0.57–1.25)	0.88 (0.55–1.40)	0.80 (0.49–1.29)
T3 (high)	277 (33.4)	44 (26.8)	0.68 (0.44–1.02)	0.81 (0.46–1.43)	0.79 (0.44–1.42)
p for trend			0.071	0.510	0.534
Fruit					
T1 (low)	276 (33.3)	72 (43.9)	1.00	1.00	1.00
T2 (medium)	277 (33.4)	55 (33.5)	0.76 (0.52–1.12)	0.80 (0.52–1.24)	0.86 (0.55–1.36)
T3 (high)	277 (33.4)	37 (22.6)	0.51 (0.33–0.78)	0.55 (0.34–0.89)	0.55 (0.33–0.92)
p for trend			0.003	0.016	0.019

Model I: crude model; model II: adjusted for age, sex, family history of gastric cancer, smoking status, regular exercise, education, occupation, income and total energy intake; model III: additionally adjusted for *H. pylori* infection status

Table S3. Interaction between GGM derived dietary patterns and sex in the risk of GC.

	Males			Females			
**Dietary pattern**	**T1 (low)**	**T2 (medium)**	**T3 (high)**	**T1 (low)**	**T2 (medium)**	**T3 (high)**	***p*-interaction**
Vegetable and seafood pattern							
No. controls/cases	180/104	179/115	181/51	97/61	96/47	97/37	
Crude OR	1.00 (ref)	1.11 (0.79–1.56)	0.49 (0.33–0.72)	1.00 (ref)	0.78 (0.49–1.25)	0.61 (0.37–0.99)	0.669
Model I OR	1.00 (ref)	1.22 (0.82–1.80)	0.51 (0.32–0.81)	1.00 (ref)	0.85 (0.50–1.46)	0.76 (0.43–1.34)	0.964
Model II OR	1.00 (ref)	1.25 (0.82–1.91)	0.55 (0.34–0.89)	1.00 (ref)	1.04 (0.58–1.84)	0.82 (0.45–1.51)	0.964
Snacks and fat							
No. controls/cases	179/99	180/100	181/71	96/55	97/60	97/30	
Crude OR	1.00 (ref)	1.00 (0.71–1.42)	0.71 (0.49–1.03)	1.00 (ref)	1.08 (0.68–1.71)	0.54 (0.32–0.91)	0.471
Model I OR	1.00 (ref)	1.07 (0.72–1.60)	0.78 (0.50–1.20)	1.00 (ref)	1.09 (0.65–1.85)	0.62 (0.31–1.22)	0.277
Model II OR	1.00 (ref)	1.03 (0.67–1.58)	0.80 (0.50–1.28)	1.00 (ref)	1.29 (0.73–2.27)	0.65 (0.32–1.34)	0.180
Meat							
No. controls/cases	180/119	180/82	180/69	97/57	96/38	97/50	
Crude OR	1.00 (ref)	0.69 (0.48–0.98)	0.58 (0.40–0.83)	1.00 (ref)	0.67 (0.41–1.11)	0.88 (0.55–1.41)	0.174
Model I OR	1.00 (ref)	0.84 (0.56–1.27)	1.17 (0.72–1.90)	1.00 (ref)	0.72 (0.40–1.30)	0.85 (0.46–1.56)	0.165
Model II OR	1.00 (ref)	0.93 (0.60–1.44)	1.23 (0.74–2.06)	1.00 (ref)	0.67 (0.36–1.27)	0.65 (0.34–1.23)	0.426
Fruits							
No. controls/cases	180/124	180/80	180/66	97/82	96/35	97/28	
Crude OR	1.00 (ref)	0.65 (0.46-0.91)	0.53 (0.37–0.77)	1.00 (ref)	0.43 (0.27–0.70)	0.34 (0.20–0.57)	0.120
Model I OR	1.00 (ref)	0.81 (0.54-1.21)	0.77 (0.50–1.17)	1.00 (ref)	0.54 (0.32–0.93)	0.56 (0.32–1.00)	0.245
Model II OR	1.00 (ref)	0.77 (0.50-1.19)	0.76 (0.48–1.19)	1.00 (ref)	0.59 (0.33–1.05)	0.62 (0.34–1.14)	0.403

Model I: crude model; model II: adjusted for age, family history of gastric cancer, smoking status, regular exercise, education, occupation, income and total energy intake; model III: additionally adjusted for *H. pylori* infection status

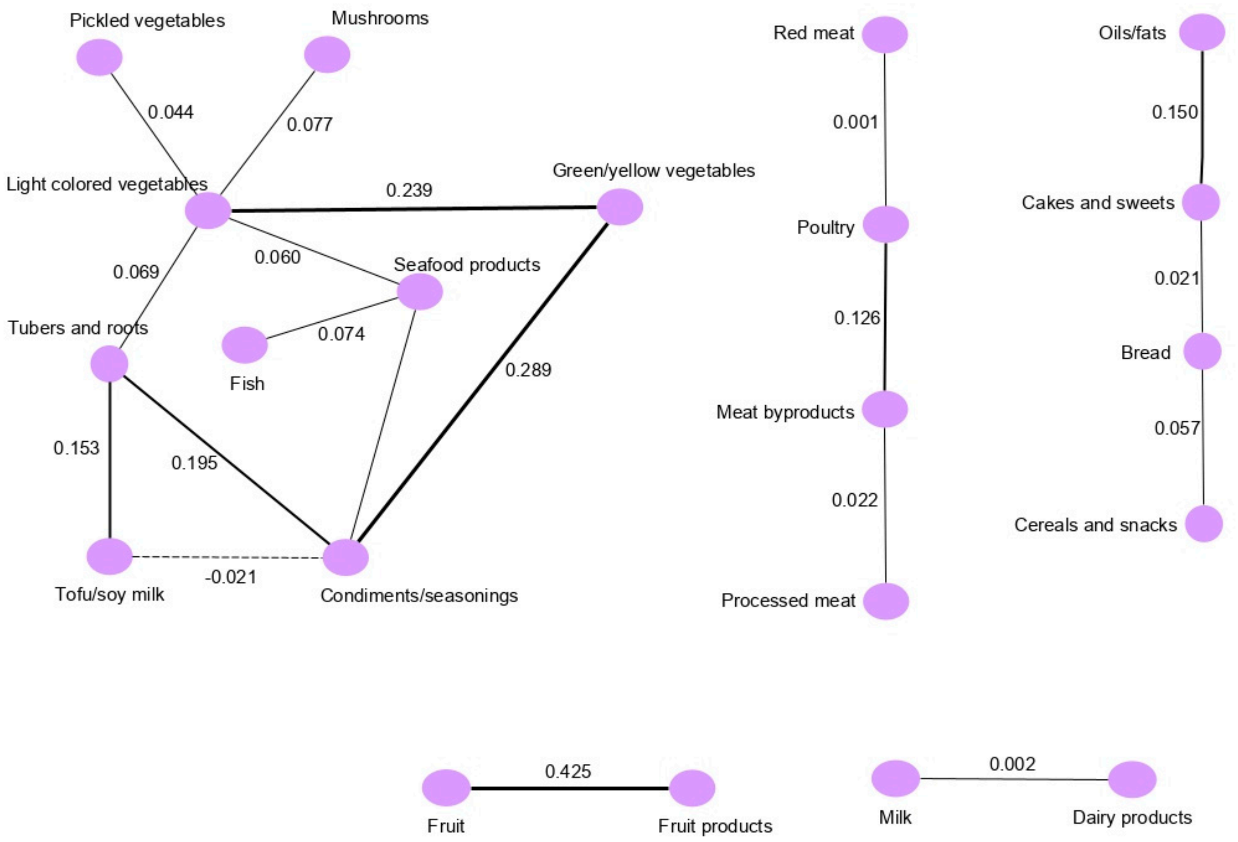

Figure S1. Dietary intake networks for intestinal type GC derived by Gaussian graphical models.

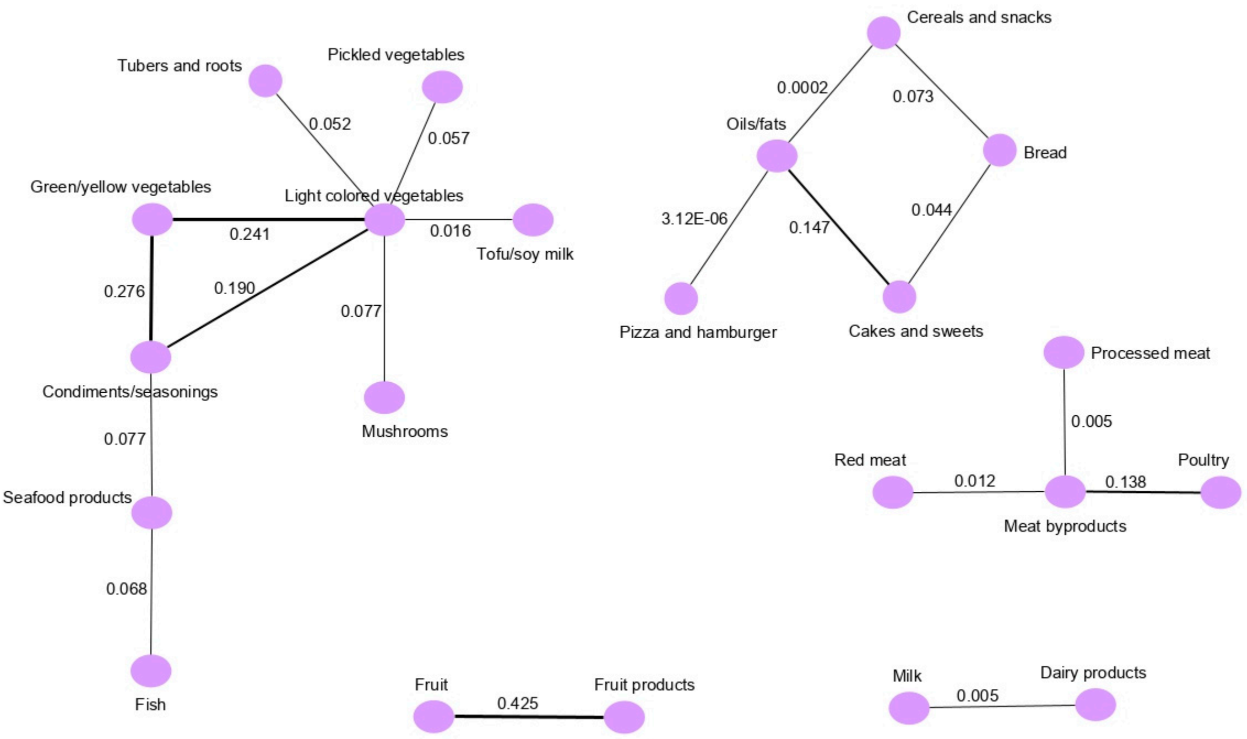

Figure S2. Dietary intake networks for diffuse type GC derived by Gaussian graphical models.
